# A novel panel of α-synuclein antibodies reveal distinctive staining profiles in synucleinopathies

**DOI:** 10.1371/journal.pone.0184731

**Published:** 2017-09-14

**Authors:** Jess-Karan S. Dhillon, Cara Riffe, Brenda D. Moore, Yong Ran, Paramita Chakrabarty, Todd E. Golde, Benoit I. Giasson

**Affiliations:** Department of Neuroscience, Center for Translational Research in Neurodegenerative Disease, and McKnight Brain Institute, College of Medicine University of Florida, Gainesville, Florida, United States of America; Louisiana State University Health Sciences Center, UNITED STATES

## Abstract

Synucleinopathies are a spectrum of neurodegenerative diseases characterized by the intracellular deposition of the protein α-synuclein leading to multiple outcomes, including dementia and Parkinsonism. Recent findings support the notion that across the spectrum of synucleinopathies there exist diverse but specific biochemical modifications and/or structural conformations of α-synuclein, which would give rise to protein strain specific prion-like intercellular transmission, a proposed model that could explain synucleinopathies disease progression. Herein, we characterized a panel of antibodies with epitopes within both the C- and N- termini of α-synuclein. A comprehensive analysis of human pathological tissue and mouse models of synucleinopathy with these antibodies support the notion that α-synuclein exists in distinct modified forms and/or structural variants. Furthermore, these well-characterized and specific tools allow the investigation of biochemical changes associated with α-synuclein inclusion formation. We have identified several antibodies of interest with diverse staining and epitope properties that will prove useful in future investigations of strain specific disease progression and the development of targeted immunotherapeutic approaches to synucleinopathies.

## Introduction

Synucleinopathies are neurodegenerative disorders, which include Parkinson’s disease (PD), multiple system atrophy (MSA), and dementia with Lewy Bodies (DLB), characterized by the presence of pathological inclusions comprised of α-synuclein (αS) [1–6]. However, the structural conformation in which αS aggregates can differ between disease states and brain regions [7–9]. In PD and DLB, nigral, neuronal αS pathology presents as round, eosinophilic, perikaryal inclusions with a very defined core and halo termed classical Lewy bodies (LB) in addition to less structured fibrillar inclusions in neuronal processes, known as Lewy neurites (LN) [2,5,6]. However, cortical LBs, present in DLB, are typically more irregular in shape and they are not laminated (i.e. they do not have a corona and core) [2,6]. In MSA, αS inclusion pathology, termed glial cytoplasmic inclusions (GCI), is predominantly present in the oligodendrocyte [2,6,10].

In recent years prion-like conformational templating has emerged as a plausible mechanism that may explain the stereotypic progression of pathogenic αS into different brain regions [5,11–13]. In addition, similar to prion diseases [14,15], it is postulated that specific αS conforms may lead to unique conformation templating of αS leading to varied but specific pathologies or protein “strains” [5,7,11–13,16]. This hypothesis poses that misfolded protein structural variance is a key trait responsible for producing the spectrum of disease states in synucleinopathies. Some recent studies suggest that the mechanism(s) involved in the intercellular transmission of αS aggregates may result from the generation of unique αS cleavage products that may specifically contribute to the spread of pathology [17,18]. Overall, these findings indicate a clear need for well characterized tools capable of assessing various forms of processed αS.

Herein we describe and characterize a novel series of αS antibodies with epitopes specific for various amino and carboxy regions of αS protein. We demonstrate that these antibodies show marked differences in their ability to recognize pathological αS inclusions in human synucleinopathies and various animal models. Such differential neuropathological attributes support the notion that subtle variations in misfolded αS protein or αS strains may serve as signatures in the spectrum of synucleinopathies.

## Materials and methods

### Production and purification of recombinant αS proteins and recombinant αS fusion proteins

All recombinant proteins were expressed in *Escherichia coli* (*E*. *coli)* BL21 (DE3)/RIL (Agilent Technologies, Santa Clara, CA). Recombinant full-length human αS, β-synuclein (βS), and γ-synuclein (γS), mouse αS and C-terminal truncated human αS corresponding to residues 1–89, 1–102, 1–110, 1–120, 1–125 and 1–130 were expressed using the respective cDNA cloned into the bacterial expression plasmid pRK172 and purified as previously described [19,20]. All other chimeric protein cDNAs were synthesized (Genscript, Piscataway, NJ, USA) and cloned into pET16b vector. Recombinant 21–140 human αS with an ATG codon added at the N-terminus and the nucleotide sequence for fifty Ala amino acid residues added after residue 140 of human αS designated protein 21–140 αS/poly 50A (used to generate 33A/S antibody series) was purified using a HiTrap Q HP column (GE Healthcare Life Sciences) with NaCl gradient elution. Recombinant 21–140 human αS with an ATG codon added at the amino-terminus and the nucleotide sequence for human Aβ1–42 added after residue 140 of human αS designated protein 21–140 αS/Aβ1–42 (used to generate 15 antibody series) was purified using a HiTrap Q HP column (GE Healthcare Life Sciences) followed by size exclusion chromatography. Recombinant 21–140 human αS with an ATG codon added at the amino-terminus and the nucleotide sequence for the first 184 amino acids of 0N human tau protein added after residue 140 of human αS designated 21–140 αS/N-term tau (used to generate 97 antibody series) was purified using a HiTrap Q HP column (GE Healthcare Life Sciences) followed by size exclusion chromatography. Recombinant 21–140 human αS with an ATG codon added at the amino-terminus followed by the nucleotide sequence for residues 244–372 in 4R human tau designated 21–140 αS/K18 (used to generate 71 and 74 antibody series) was purified using a HiTrap SP column (GE Healthcare Life Sciences) with NaCl gradient elution flowed by size exclusion column. Recombinant 21–140 human αS with an ATG codon added at the amino-terminus followed by the nucleotide sequence for residues 244–372 in 4R human tau followed by human Aβ1–42 designated 21–140 αS/K18/Aβ1–42 (used to generate 94 series) was purified using a HiTrap Q HP column (GE Healthcare Life Sciences) followed by size exclusion chromatography. Protein concentrations were determined by bicinchoninic acid (BCA) assay using bovine serum albumin (BSA; Pierce, Rockford, IL) as a standard.

### Generation of new mouse monoclonal antibodies

All procedures were performed according to the NIH Guide for the Care and Use of Experimental Animals and were approved by the University of Florida Institutional Animal Care and Use Committee.

To generate antibodies targeted to the amino-terminus of human αS, the peptide (CDVFMKGLSKAKEGVVAAAEK) corresponding to amino acids 2–21 in human αS with an added Cys residue was synthesized and purified by GenScript (Piscataway, NJ). The lyophilized peptides were reconstituted in phosphate buffered saline (PBS) and conjugated to Imject maleimide-activated mariculture keyhole limpet hemocyanin (mcKLH; Thermo Scientific, Waltham, MA).

Female BALB/c mice (Jackson Laboratory, Bar Harbor, ME) were used for immunization with full-length human αS, 21–140 αS/poly 50A (33A/S antibody series), 21–140 αS/Aβ1–42 (15 antibody series) and 2–21 αS/KLH while C3H/C57BL6 mice were used for immunization with 21–140 αS/K18 (71 and 74 antibody series), 21–140 αS /Aβ1–42 (94 antibody series) and 21–140 αS /N-term tau (97 antibody series). Proteins (100 μg) in 200 μl phosphate buffered saline (PBS) were emulsified with 100 μl of either Freunds complete adjuvant (1^st^ injection; Sigma Aldrich, St. Louis, MO) or Freunds incomplete adjuvant (subsequent injections; Sigma Aldrich, St. Louis, MO). For the first immunization, mice were injected subcutaneously. An intraperitoneal (IP) injection was administered 3 weeks later. Six weeks following the initial injection, mice were boosted with an IP injection of the proteins in PBS. Three days later, mice were euthanized by CO_2_ inhalation and spleens were harvested using aseptic technique.

Mouse myeloma (Sp2/O-Ag14; ATCC, Manassas, VA) cells were maintained in high glucose (4.5gm/L) Dulbecco’s Modified Eagle Medium (DMEM) with 10% NCTC 135 Media (Sigma Aldrich, St. Louis, MO), 20% hybridoma grade fetal bovine serum (FBS; Hyclone, Logan, UT), 100 U/ml penicillin, 100 U/ml streptomycin, 2 mM L-glutamine, 0.45 mM pyruvate, 1 mM oxaloacetate, and 0.2 U/ml insulin at 37°C and 8% CO_2_. Spleens were gently homogenized in 5% FBS/Hank’s balanced salt solution (HBSS; Lonza, Walkersville, MD) and centrifuged to pellet cells. The cell pellet was resuspended in red blood cell lysis buffer (Sigma Aldrich, St. Louis, MO) and diluted with HBSS after one minute. The cells were then washed twice by centrifuging at 100 x g for 10 minutes and resuspending in HBSS. Sp2/O-Ag14 cells were also washed twice with HBSS. Five million Sp2/O-Ag14 cells were added to 50 million spleen cells and after centrifuging at 100 x g for 10 minutes onto a culture dish, fusion was induced with 50% polyethylene glycol 1450 (Sigma Aldrich, St. Louis, MO). After washing with HBSS, cells were incubated in Sp2/O-Ag14 media at 37°C with 8% CO_2_ overnight. The next day, the cells were gently detached from the plate and distributed into 96 well plates with Sp2/O-Ag14 media/0.5% hybridoma enhancing supplement (Sigma Aldrich, St. Louis, MO)/HAT selection supplement (Sigma Aldrich, St. Louis, MO).

### Hybridoma screening

All hybridoma clones were screened for reactivity to αS by enzyme-linked immunosorbent assay (ELISA). MaxiSorp plates (Thermo Scientific, Waltham, MA) or Immulon 4HBX plates (ThermoFisher Scientific, Waltham, MA) were coated with 1 μg/ml recombinant human αS in PBS or 100 mM sodium bicarbonate overnight at 4°C and blocked with 5% FBS/PBS or 1% Block ACE (Bio-Rad) in PBS the following day for 1 hour. Media from the hybridomas were applied to plates, which were then incubated at room temperature for 3 hours. The plates were then washed with PBS, and incubated with goat anti-mouse secondary antibody conjugated to horse radish peroxidase (HRP; Jackson Immuno Research Labs, West Grove, PA) for 1 hour at room temperature. Detection was completed using TMB substrate untilcolor change was observed (Pierce, Rockford, IL). Reactions were then quenched with 1M HCl and absorbance was measured at 450 nm.

Further characterization of clones of interest utilized ELISA with plates coated with recombinant human αS, βS or γS or mouse αS. In addition, clones were screened for epitope specificity utilizing C-terminal truncated constructs of human αS of variable lengths: 1–89, 1–102, 1–110, 1–120, 1–125, or 1–130. Immunotyping of clones was also completed through ELISA using Mouse Monoclonal Antibody Isotyping Reagents (Sigma Aldrich, St. Louis, MO).

### αS transgenic mice and αS knock-out mice

αS transgenic mice expressing wild type human αS (line M20) or A53T human αS (line M83) were previously described [21]. Previously generated αS knock-out (KO) mice [22] were obtained from Jackson Laboratory (Bar Harbor, ME). Homozygous M83 mice (M83^+/+^) intrinsically develop an age-dependent, progressive, severe motor phenotype leading to paralysis associated with the accumulation of CNS αS inclusion pathology [21]. Hemizygous M83 mice (M83^+/-^), which are otherwise normal, can be induced to develop CNS αS inclusion pathology by intracerebral injection of preformed fibrillar αS fibrils. Intramuscular (IM) injection of preformed αS fibrils in M83^+/-^ mice also leads to induction of αS pathology and motor impairment [23–26].

### Sequential biochemical fractionation of mouse nervous tissue

Mouse nervous tissues were biochemically fractionated as described by Emmer et al 2011 [27]. Briefly, mice were humanely euthanized by CO_2_ inhalation, and the brain, brainstem and spinal cord were harvested. The brainstem/spinal cord were dissected and placed in separate tubes. Tissues were frozen at -80°C and thawed on ice on day of processing.

Nervous tissue was homogenized with 3 volumes per gram of tissue with high salt (HS) buffer (50 mM Tris, pH 7.5, 750 mM NaCl, 20 mM NaF, 5 mM EDTA) containing protease inhibitor cocktail (1 mM phenylmethylsulfonyl and 1 mg/ml each of pepstatin, leupeptin, N-tosyl-L-phenylalanyl chloromethyl ketone, N-tosyl-lysine chloromethyl ketone and soybean trypsin inhibitor). The HS tissue homogenates then underwent sedimentation at 100,000 x g for 20 minutes and the supernatants were saved as the HS fraction. Pellets were resuspended in 3 volumes per gram of tissue with HS buffer with 1% Triton X-100 (HS/T buffer) and centrifuged at 100, 000 x g for 20 minutes. The supernatants were saved as the HS/T fraction. The pellets were then homogenized in 3 volumes per gram of tissue with HS buffer with 1 M sucrose and centrifuged at 100,000 x g for 20 minutes to float the myelin, which was discarded. Pellets were homogenized in 2 volumes per gram of tissue with radioimmunoprecipitation assay (RIPA) buffer (50 mM Tris, pH 8.0, 150 mM NaCl, 5 mM EDTA, 1% NP-40, 0.5% sodium deoxycholate, 0.1% SDS) containing protease inhibitors and centrifuged at 100,000 x g for 20 minutes. Supernatants were saved as the RIPA fraction. Pellets were then homogenized in 1 volume per gram of tissue with 2% SDS/4 M urea by probe sonication and saved as the SDS/U fractions. Protein concentrations of all fractions were determined by BCA assay using bovine serum albumin as the standard. SDS sample buffer was added to sequential fractions which were incubated for 10 minutes at 100°C for the HS and HS/T fractions or at room temperature for SDS/U fraction, and then frozen at -80°C. Equal amounts of protein (20 μg for HS and HS/T, and 10 μg for SDS/U fractions) were resolved by SDS-PAGE and analyzed by immunoblot.

For total unfractionated samples, tissue was homogenized in 4% SDS with a cocktail of protease inhibitors, probe sonicated, and solubilized by incubation for 10 minutes at 100°C. Protein concentrations were determined by BCA assay using bovine serum albumin as the standard.

### Immunoblotting analyses

Protein samples were resolved by electrophoresis on 15% polyacrylamide gels, then electrophoretically transferred to nitrocellulose membranes. Membranes were blocked with 5% milk in Tris-buffered saline (TBS) for 1 hour, then incubated overnight at 4°C with primary antibodies diluted in 5% milk/TBS. Following washing with TBS, blots were incubated with HRP conjugated goat anti-mouse or goat anti-rabbit secondary antibodies (Jackson Immuno Research Labs, West Grove, PA) diluted in 5% milk/TBS for 1 hour. Following washing with TBS, protein bands were visualized using Western Lightning-Plus ECL reagents (PerkinElmer, Waltham, MA) and images were captured using the GeneGnome XRQ system and GeneTools software (Syngene, Frederick, MD).

### Immunohistochemistry

Ethanol (70% ethanol/150 mM NaCl) or formalin fixed tissue from αS transgenic mice were utilized and are summarized in Table 1. Harvesting, fixation, and processing were conducted as previously described [24]. Paraffin embedded, formalin fixed human brain tissue was obtained through the University of Florida Neuromedicine Human Brain Tissue Bank. Sequential tissue sections were deparaffinized with xylenes, and sequentially rehydrated with graded ethanol solutions (100–70%). Antigen retrieval was performed by incubating sections in 0.05% Tween-20 in a steam bath for 60 minutes. Endogenous peroxidase activity was quenched with 1.5% hydrogen peroxide/0.005% Triton-X-100/PBS for 20 minutes. Sections were blocked with 2% FBS/0.1 M Tris, pH 7.6 then incubated with primary antibody overnight at 4°C. Following washing with 0.1 M Tris, pH 7.6, sections were incubated with biotinylated horse anti-mouse IgG (Vector Laboratories, Burlingame, CA), or biotinylated goat anti-mouse IgA (Southern Biotech, Birmingham, AL) for antibodies 1F11 and 4C5, diluted in 2% FBS/0.1 M Tris pH 7.6 for 1 hour. Sections were then washed with 0.1 M Tris, pH 7.6, and incubated with streptavidin-conjugated HRP (Vectastain ABC kit; Vector Laboratories, Burlingame, CA) diluted in 2% FBS/0.1 M Tris pH 7.6 for 1 hour. Sections were washed with 0.1 M Tris, pH 7.6, and then colorimetric development was completed using 3, 3’diaminobenzidine (DAB kit; KPL, Gaithersburg, MD). Reactions were stopped by immersing the slides in 0.1 M Tris, pH 7.6, and sections were counterstained with Mayer's hematoxylin (Sigma Aldrich, St. Louis, MO). Slides were dehydrated with an ascending series of ethanol solutions (70%-100%) followed by xylenes, and coverslipped using cytoseal (Thermo Scientific, Waltham, MA). Inclusion pathology was observed and qualitatively assessed by two independent observers.

**Table 1:** Summary of M83 transgenic mice used for immunohistochemistry studies.

M83 mouse genotype	Synucleinopathy Model	Fixation	References
C57BL6/C3H M83^+/+^	Aged 13 months	Ethanol	[21]
C57BL6/C3H M83^+/-^	Bilateral hind limb injected with mouse αS	Formalin	[26]
C57BL6/C3H M83^+/-^	Bilateral hind limb injected with mouse αS	Ethanol	[25]
C57BL6/C3H M83^+/-^	Bilateral hippocampal injected with human αS fibrils	Ethanol	[24]

## Results

### Characterization of a series of novel monoclonal antibodies against αS

**Fig 1:**
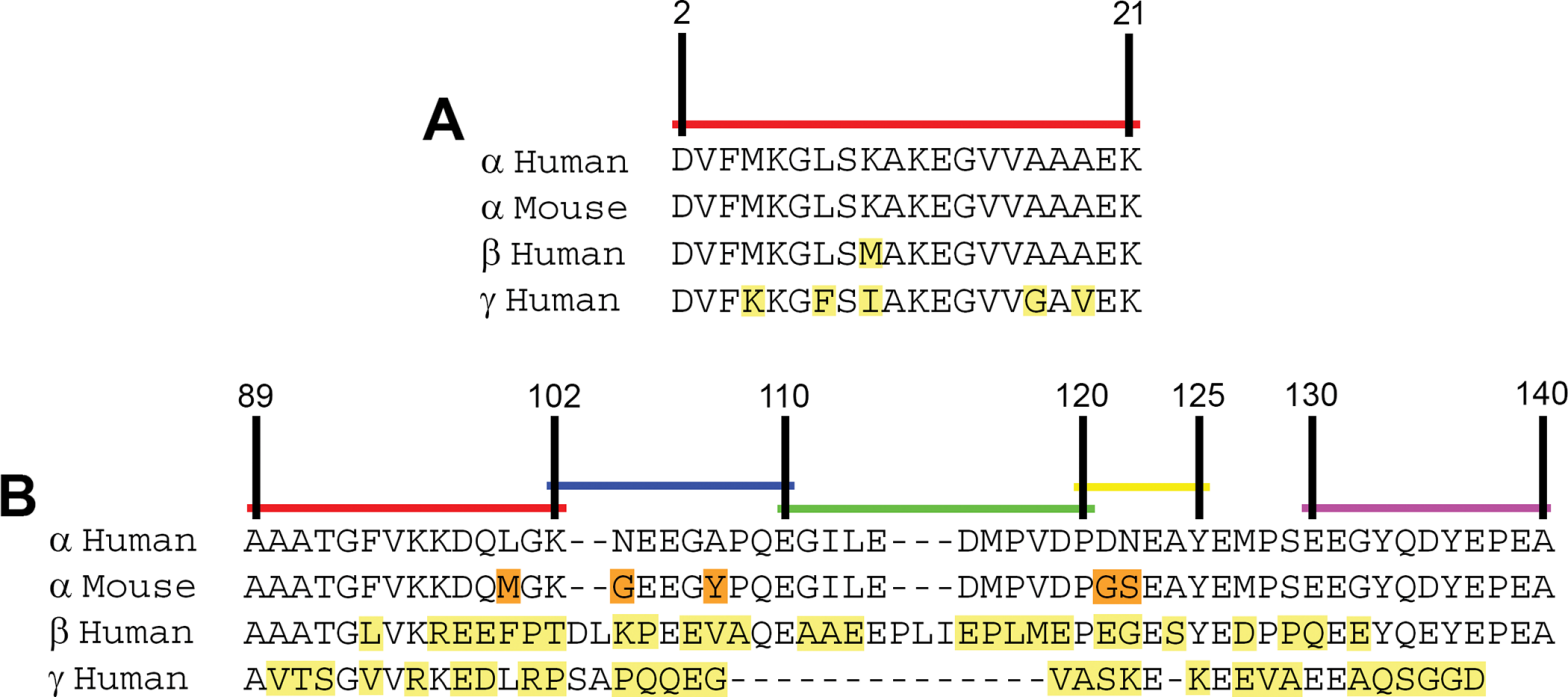
Alignment of the amino- and carboxy-terminal amino acid sequences of human and mouse αS and compared to human βS and γS. **(A)** Amino acid sequences of residues 2–21 in human αS, βS and γS and mouse αS. **(B)** Carboxy-terminal region sequence including amino acid residues 89–140 of human and mouse αS relative to the sequence of human βS and γS. Residues highlighted in orange indicate differences between human and mouse αS, while residues highlighted in yellow depict differences between human synuclein proteins.

**Fig 2:**
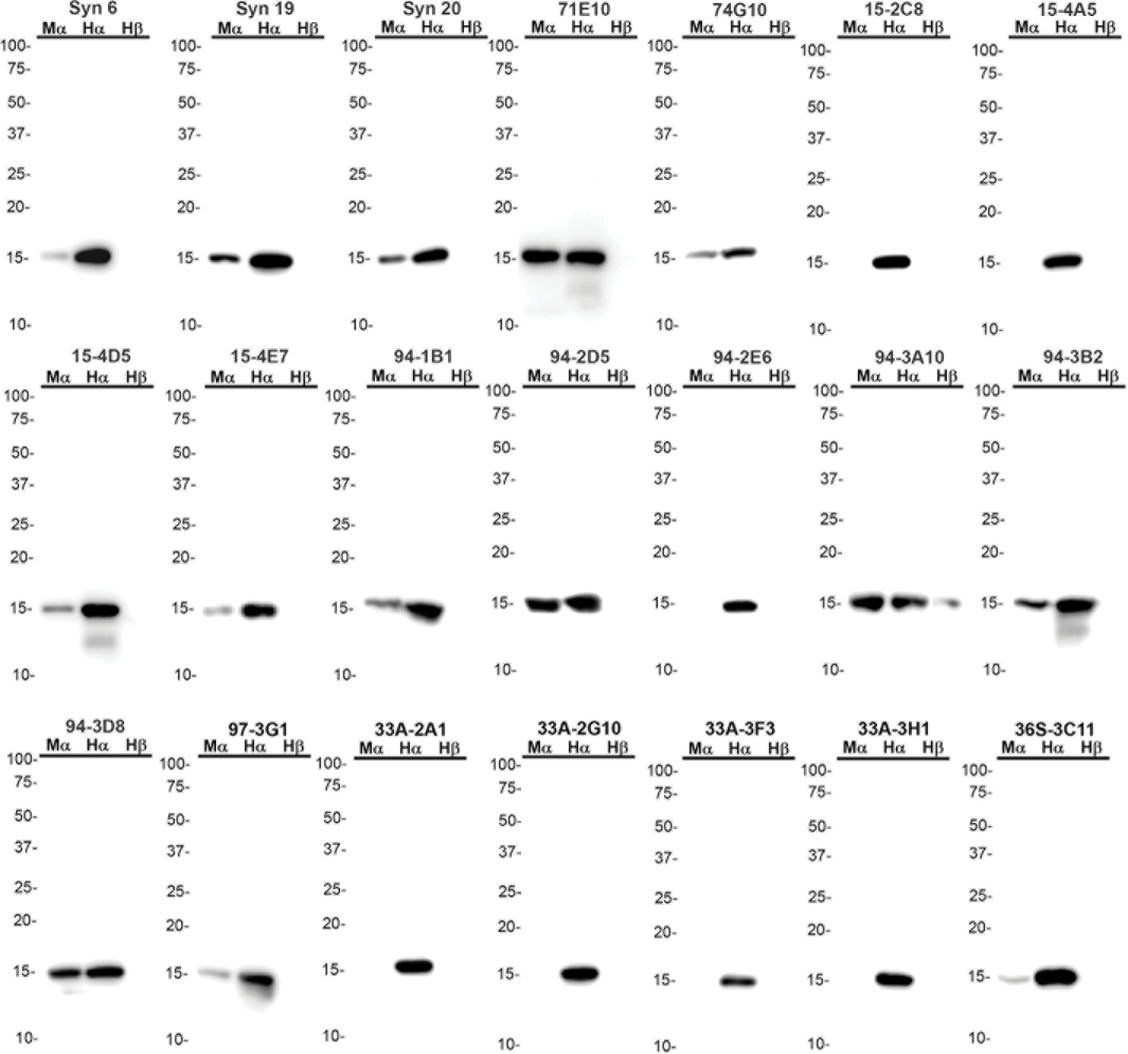
Immunoblotting analysis of epitope reactivity of anti-αS antibodies using recombinant mouse αS, human αS, and human βS. 100 ng of each recombinant protein was loaded on 15% polyacrylamide gels and analyzed as described in “Materials and Methods”. Immunoblot analysis was performed with each antibody indicated above. The mobilities of molecular mass markers are indicated on the left. Mα = Mouse αS, Hα = Human αS, Hβ = Human βS.

We generated a series of new αS reactive monoclonal antibodies either by directly targeting αS as the immunogen or using αS as a carrier to try to generate antibodies to other polypeptides, but that also yielded antibodies to αS (summarized in Table 2). The specificity of each antibody was initially determined through ELISA screening against recombinant human and mouse αS as well as human βS and γS (Table 2). The isotype of each monoclonal antibody was determined and the epitope recognized by each antibody was assessed using a series of carboxy-truncated recombinant human αS proteins (Fig 1 and Table 2). The αS antibodies generated against recombinant forms of αS mapped within residues 89–140 of αS (Table 2). The reactivity of these antibodies towards human and mouse αS and human βS protein was also confirmed by immunoblotting (Fig 2). None of these antibodies reacted with γS (Table 2) consistent with the limited homology between the carboxy-terminal region of αS and γS (Fig 1). Most antibodies did not react with βS, but antibody 94-3A10, with an epitope to residues 130–140 which is relatively conserved in βS, reacted with βS by both ELISA and immunoblotting (Table 2 and Fig 2). Several antibodies were also human αS specific, especially those that map to residues 120–125 (Table 2 and Fig 2), which has 2 amino acid differences between human and mouse αS (Fig 1). Several antibodies (Syn 19, Syn 20, and 94-2D5), target residues 89–102. This region of αS is conserved between human and mouse αS except for one amino acid (Fig 1), which can explain the weaker relative reactivity for Syn 19 and Syn 20 to mouse αS compared to human αS.

**Table 2:** Summary of specificity and epitopes recognized by the novel αS antibodies.

Antibody	Antigen Used	Human αS	Mouse αS	Human βS	Human γS	Epitope	Isotype
**Syn 6**	αS	yes	yes	no	no	110–120	IgG_1_
**Syn 19**	αS	yes	weak	no	no	89–102	IgG_1_
**Syn 20**	αS	yes	yes	no	no	89–102	IgG_1_
**71E10**	αS 21-140/K18	yes	yes	no	no	110–120	IgG_1_
**74G10**	αS 21-140/K18	yes	yes	no	no	110–120	IgG_1_
**15-2C8**	αS 21-140/Aβ1–42	yes	no	no	no	120–125	IgG_1_
**15-4A5**	αS 21-140/Aβ1–42	yes	no	no	no	120–125	IgG_1_
**15-4D5**	αS 21-140/Aβ1–42	yes	yes	weak	no	102–110	IgG_1_
**15-4E7**	αS 21-140/Aβ1–42	yes	yes	weak	no	130–140	IgG_1_
**94-1B1**	αS 21-140/K18/Aβ1–42	yes	yes	no	no	110–120	IgG_2A_
**94-2D5**	αS 21-140/K18/Aβ1–42	yes	yes	no	no	89–102	IgG_2A_
**94-2E6**	αS 21-140/K18/Aβ1–42	yes	no	no	no	102–110	IgG_2A_
**94-3A10**	αS 21-140/K18/Aβ1–42	yes	yes	yes	no	130–140	IgG_1_
**94-3B2**	αS 21-140/K18/Aβ1–42	yes	yes	no	no	102–110	IgG_1_
**94-3D8**	αS 21-140/K18/Aβ1–42	yes	yes	no	no	110–120	IgM
**97-3G1**	αS 21-140/N-term tau	yes	yes	weak	no	102–110	IgG_2B_
**33A-2A1**	αS 21-140/poly 50A	yes	no	no	no	120–125	IgM
**33A-2G10**	αS 21-140/poly 50A	yes	no	no	no	120–125	IgG_1_
**33A-3F3**	αS 21-140/poly 50A	yes	no	no	no	120–125	IgG_2B_
**33A-3H1**	αS 21-140/poly 50A	yes	no	no	no	120–125	IgG_1_
**36S-3C11**	αS 21-140/poly 50A	yes	yes	no	no	110–120	IgG_1_
**1D12**	2–21 αS peptide	yes	yes	no	no	2–21	IgG_1_
**1F11**	2–21 αS peptide	yes	yes	weak	no	2–21	IgA
**2H6**	2–21 αS peptide	yes	yes	yes	yes	2–21	IgG_1_
**4B10**	2–21 αS peptide	yes	yes	yes	yes	2–21	IgG_1_
**4C5**	2–21 αS peptide	yes	yes	yes	yes	2–21	IgA
**9C10**	2–21 αS peptide	yes	yes	weak	no	2–21	IgG_1_

Reactivity to human αS, mouse αS, human βS, and human γS was assessed by ELISA as described in “Materials and Methods”. Epitope mapping for the antibodies generated using recombinant αS was determined by ELISA with full-length (i.e. 1–140) and 1–89, 1–102, 1–110, 1–120, 1–125 and 1–130 recombinant human αS.

**Fig 3:**
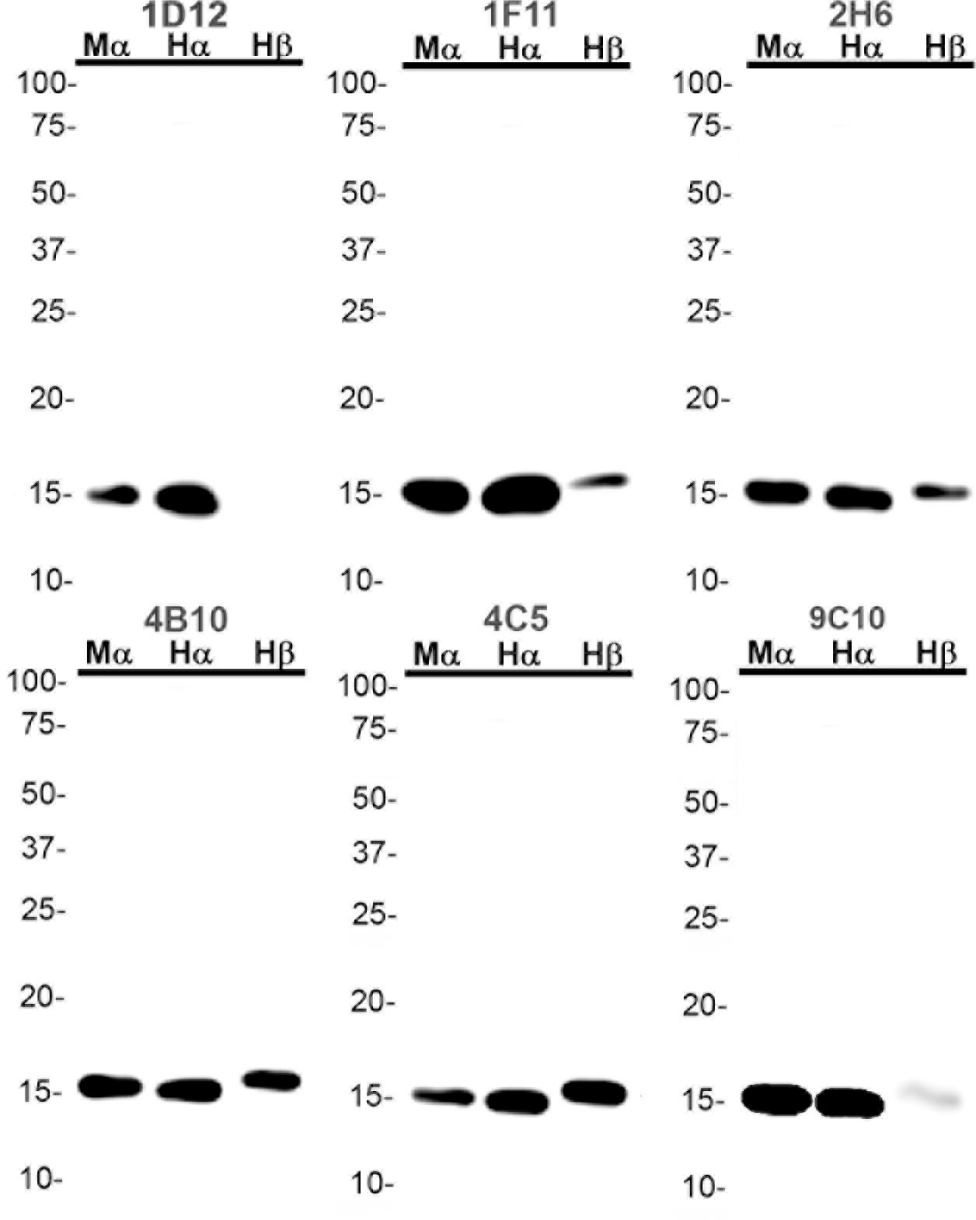
Immunoblotting analysis of epitope reactivity of N-terminal anti-αS antibodies using recombinant mouse αS, human αS, and human βS. 100 ng of each recombinant protein were loaded on 15% polyacrylamide gels and analyzed as described in “Material and Methods”. Immunoblot analysis was performed with each antibody indicated above. The mobilities of molecular mass markers are indicated on the left. Mα = Mouse αS, Hα = Human αS, Hβ = Human βS.

Since previous studies indicate that amino-terminal αS antibodies can have unique properties at preferentially recognizing αS pathology [28,29], an additional set of antibodies was generated to specifically target this region by immunizing with a peptide corresponding to residues 2 through 21 in αS (Table 2). All of the N-terminal antibodies generated by this strategy could react with human and mouse αS and all except 1D12 bound human βS, to some extent. (Table 2 and Fig 3). 1F11 and 9C10 only weakly bound human βS and did not react with human γS at all, while the other four N-terminal antibodies reacted with human γS (Table 2 and Fig 3). Consistent with this data, within αS residues 2 through 21, only one amino acid differs between human αS and βS at residue 10 (Fig 1), which seems to be necessary for the binding of 1D12.

**Fig 4:**
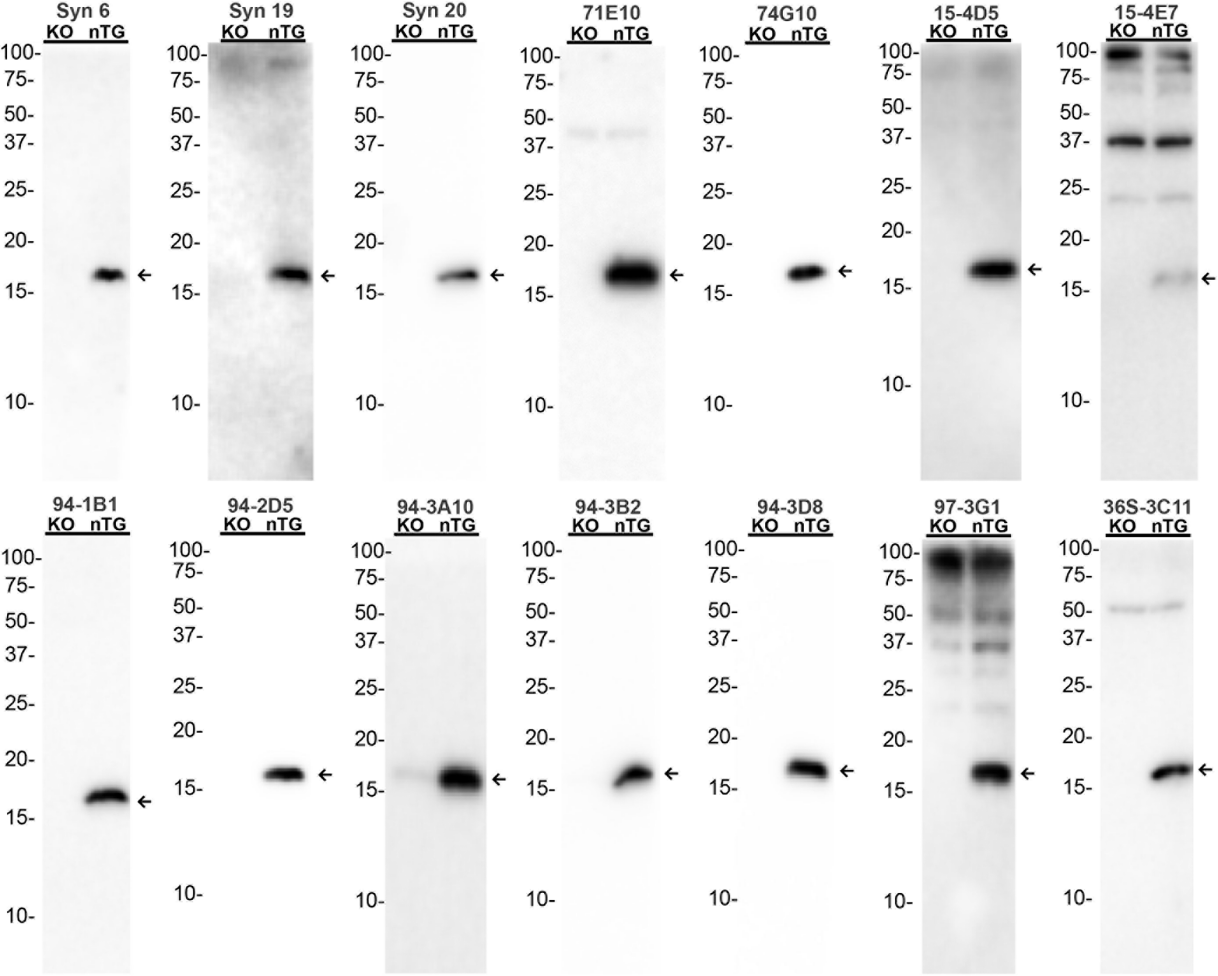
Immunoblotting analysis of various αS antibodies that react with mouse αS using total brain lysates. 20 μg of total brain homogenates from αS KO and nTG mice were resolved on 15% polyacrylamide gels and analyzed as described in “Materials and Methods”. Immunoblot analysis was performed with each antibody indicated above. The arrows indicates αS. The mobilities of molecular mass markers are indicated on the left. Both αS KO and nTG mice are of the same genetic background as experimental synucleinopathy models, C57BL6/C3H.

**Fig 5:**
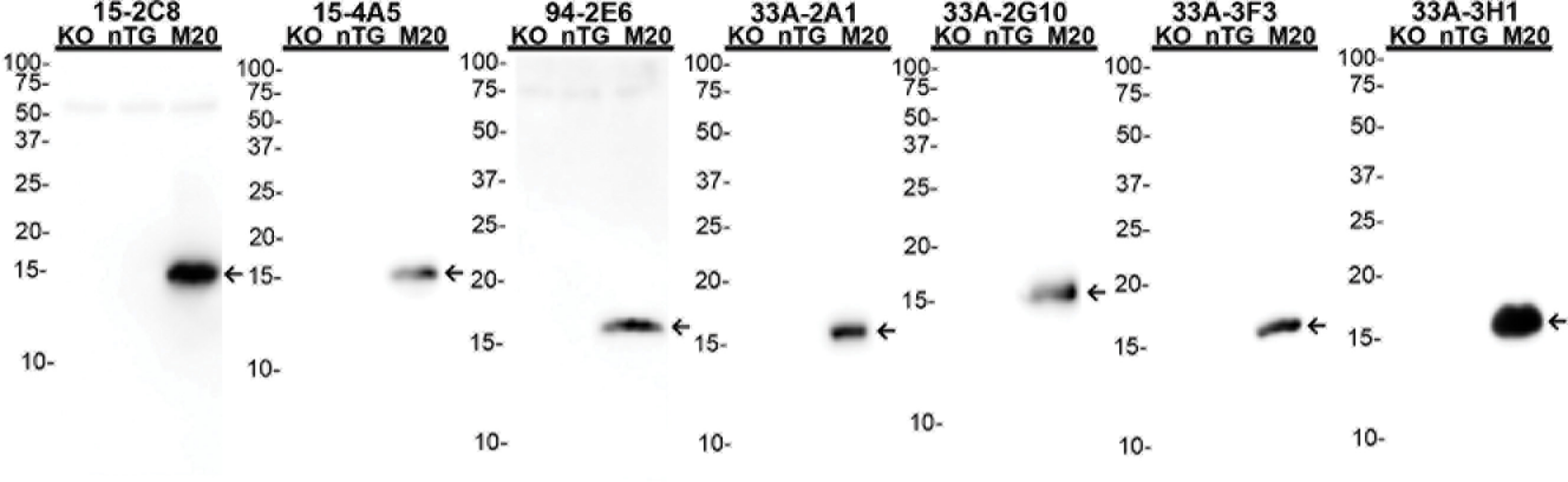
Immunoblotting analysis of various αS antibodies specific for human αS using total brain lysates from M20 human αS transgenic mice. 20 μg of total brain homogenates from αS KO, nTG mice and M20 human αS transgenic mice were resolved on 15% polyacrylamide gels and analyzed as described in “Materials and Methods”. Immunoblot analysis was performed with each antibody indicated above. The arrows indicates human αS. The mobilities of molecular mass markers are indicated on the left.

The specificities of the C-terminal antibodies was assessed by immunoblotting using total mouse brain homogenates from αS KO and nTG mice for the antibodies that could react with mouse αS (Fig 4). For the antibodies that are human αS specific, we also included homogenates from M20 human αS transgenic mice that overexpress human WT αS (Fig 5). Most of the antibodies that reacted with mouse αS demonstrated little cross-reactivity with other non-αS proteins in lysates from αS null mice. However, 15-4E7 and 97-3G1 showed non-specific interaction with several non-αS proteins and 94-3A10 could still detect βS in lysate from αS null mice (Fig 4). Similarly human αS specific antibodies demonstrated scant cross-reactivity to other non-αS proteins (Fig 5).

**Fig 6:**
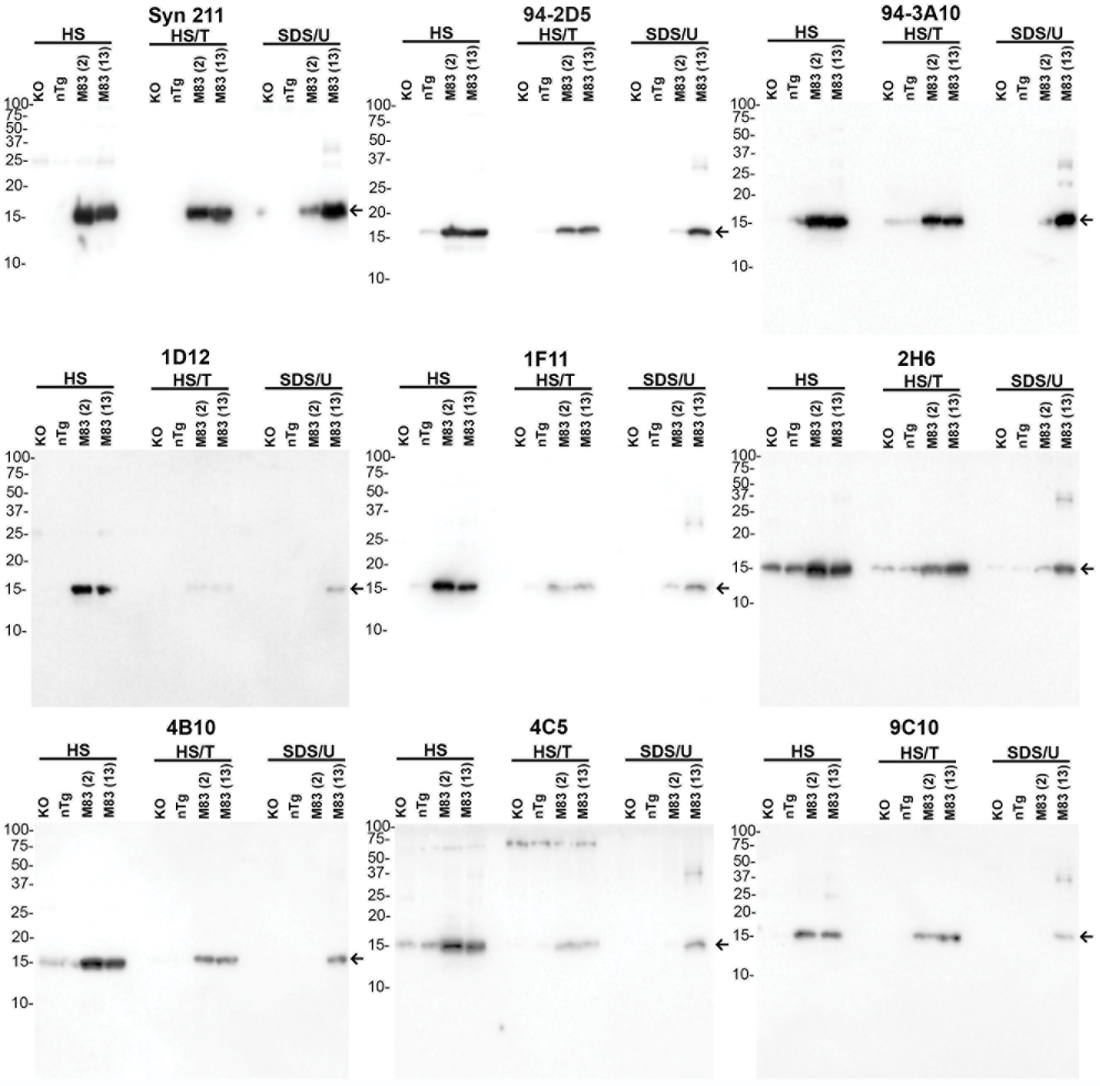
Immunoblotting analysis using sequentially fractionated mouse nervous tissue using various novel αS antibodies. The brainstem and spinal cord of an αS KO, a nTG, a 2-month old unaffected homozygous M83 mice, and a 13-month old motor impaired homozygous M83 mice were sequentially fractionated as described in “Materials and Methods”. 20 μg from the high salt (HS) and high salt/Triton (HS/T) and 10 μg from the SDS/urea (SDS/U) fractions were loaded on 15% polyacrylamide gels and analyzed with various antibodies as described above each blot. The arrows indicates αS. The mobilities of molecular mass markers are indicated on the left.

Immunoblotting analysis was expanded for the N-terminal antibodies and some of the C-terminal antibodies (94-2D5 and 94-3A10) using sequentially fractionated mouse brain tissue, a method that can be used to monitor the presence of αS inclusion pathology (Fig 6). Brain homogenates from KO, nTG, young M83 (2 months old), and aged M83 (13 months old) mice were utilized. Whereas M20 transgenic mice express wild type human αS transgene, M83 transgenic mice express A53T human αS, a mutation that causes PD [21]. Homozygous M83 mice develop age-dependent αS inclusion pathology that accumulate in the detergent insoluble, SDS/urea soluble fraction [21]. A previously characterized antibody specific for human αS, Syn211, was included for comparison [30]. All the antibodies analyzed were relatively specific for αS monomer in the soluble cell fraction and further revealed the accumulation of detergent insoluble αS in aged M83 mice. As expected antibodies 2H6, 4B10, and 4C5 could still react with a protein with a similar mass as αS in the high-salt fraction of αS null mice, which is due to the ability of these antibodies to cross-react with βS. 4C5 reacted with an ~70 kDa non-αS protein predominantly in the HS/T-soluble fraction (Fig 6).

### Immunohistochemical characterization of αS inclusion pathology using the new series of anti-αS antibodies

**Fig 7:**
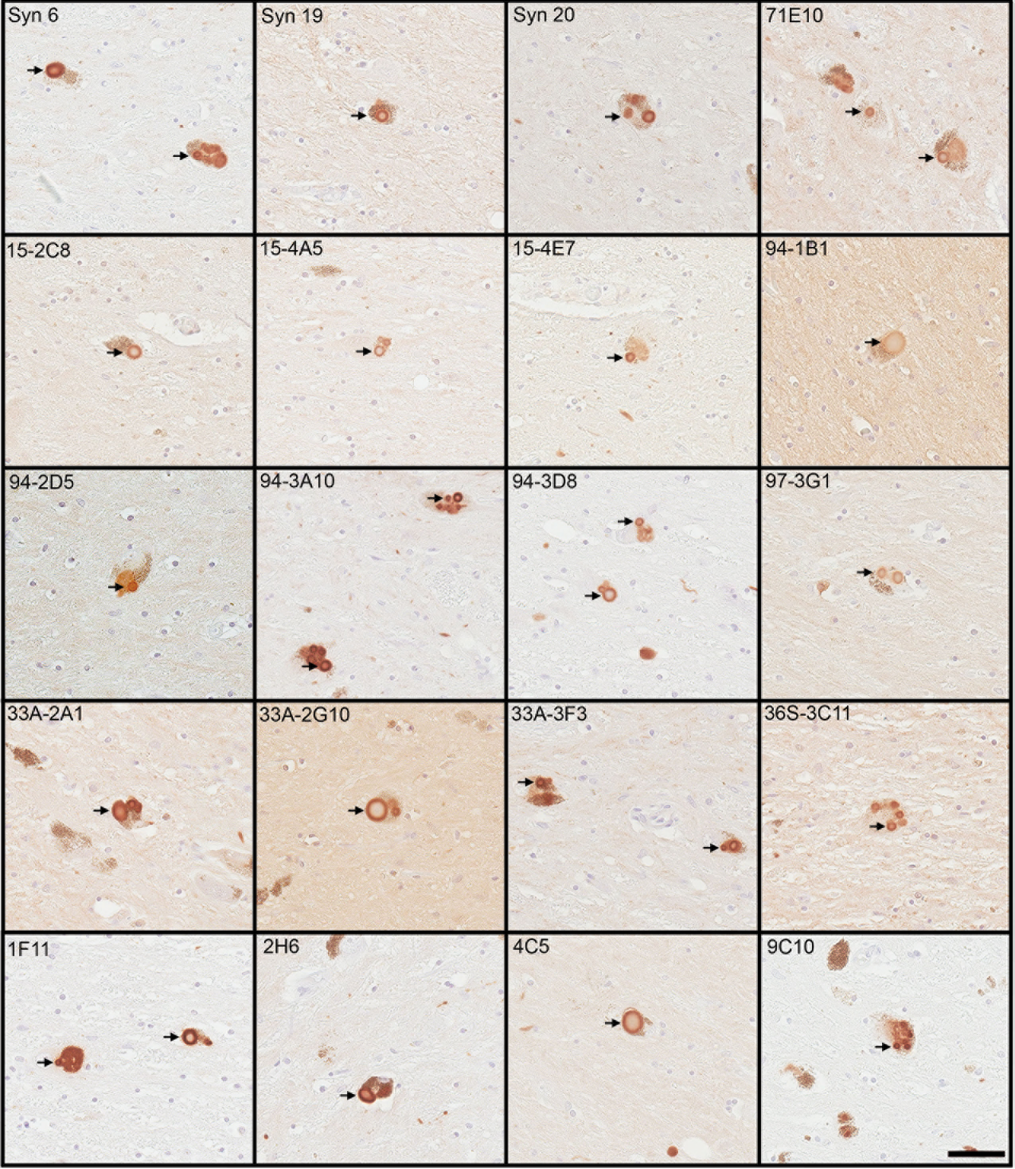
Representative immunohistochemistry depicting Lewy bodies stained with an array of αS antibodies within neuromelanin laden midbrain neurons of PD patients. Tissues sections were immunostained with each indicated antibody and developed with DAB as described in “Material and Methods” and counterstained with hematoxylin. Arrows indicate LBs. Bar = 50 μm.

**Fig 8:**
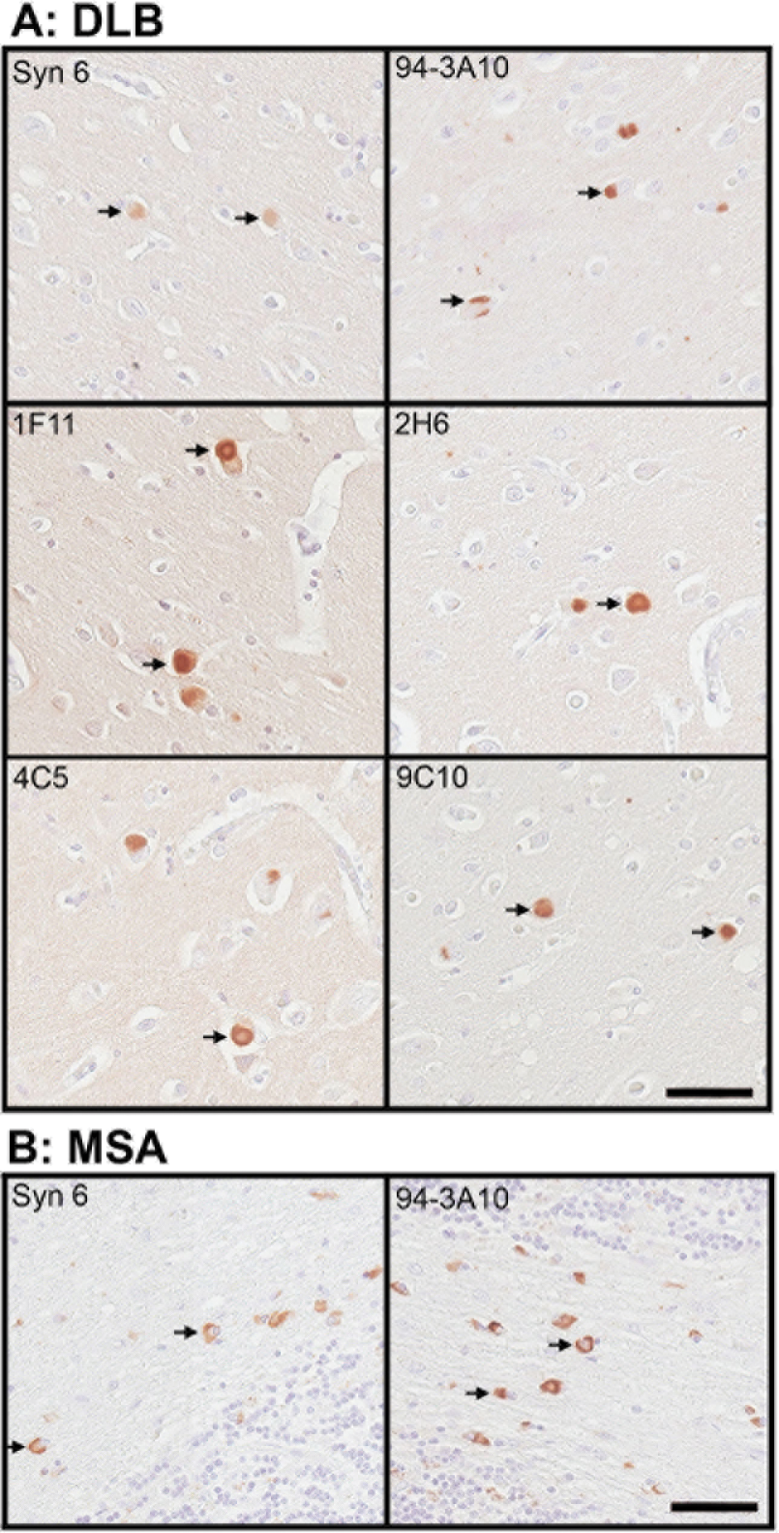
Representative immunohistochemistry of the cingulate gyrus of a DLB patient, and the cerebellum of a MSA patient stained with the indicated antibodies. Tissues sections from the cingulate cortex of a DLB patient (A) and the cerebellum of an MSA patient (B) were immunostained with each indicated antibody and developed with DAB as described in “Material and Methods and counterstained with hematoxylin. Arrows indicate cortical LBs in A and GCIs in B. Bar = 50 μm.

Immunohistochemistry was performed on post mortem brain tissue from patients with synucleinopathies to compare the detection of αS inclusion pathology with our series of novel monoclonal antibodies. We included the substantia nigra of patients with PD and DLB, as this region contains classical LBs within dopaminergic, neuromelanin-laden neurons. We also included the cingulate cortex from DLB patients, which typically has robust cortical LBs, and the pons and cerebellum of MSA patients which have abundant GCIs [8]. Most antibodies generated were proficient in labelling nigral LBs in PD and cortical LBs in DLB patients (Fig 7 and Table 3). Staining of cortical Lewy pathology was generally less robust but C-terminal antibody 94-3A10 and all six N-terminal specific antibody yielded robust reactivity (Fig 8 and Table 3). However, the relative GCI labeling profile of this series of antibodies was different from that of Lewy pathology (Table 3). All six N-terminal specific antibodies only modestly labelled GCIs (Table 3). Syn 6, 94-3D8, and 33A-3F3 could detect GCIs moderately well while 94-3A10 proved to be the most proficient antibody, capable of targeting pathology in every human case, across each brain region investigated (Fig 8 and Table 3).

**Table 3:** Summary of the relative reactivity of αS inclusion pathology in human synucleinopathies and αS M83 mouse models with the new array of αS antibodies.

Antibody Name	PD SN	DLB SN	DLB Cing	MSA Pons	MSA CB	M83^+/-^ mouse ethanol-fixed	M83^+/-^ mouse formalin-fixed
**Syn 6**	++	+++	+	+++	+	++	+/-
**Syn 19**	++	++	+	+	+/-	+/-	+/-
**Syn 20**	++	++	+	++	+	+	+
**71-E10**	++	++	+	+/-	+/-	+/-	-
**74-G10**	+	+	+	+	+	+/-	+/-
**15-2C8**	+	+	+/-	+/-	+/-	-	-
**15-4A5**	++	++	+	+/-	+/-	+/-	-
**15-4D5**	+/-	+/-	+/-	-	-	-	-
**15-4E7**	+	+	+	+	+	+++	+++
**94-1B1**	+/-	+/-	+/-	+/-	-	-	-
**94-2D5**	+	++	+/-	-	-	+/-	+/-
**94-2E6**	+/-	+/-	+/-	+/-	+/-	+/-	+/-
**94-3A10**	++++	++++	+++	++++	+++	++	++
**94-3B2**	+	+++	+/-	-	-	+/-	-
**94-3D8**	++++	++	++	++	++	+	+
**97-3G1**	+	+	+/-	+/-	+/-	-	-
**33A-2A1**	++	++	+	+	+/-	-	-
**33A-2G10**	++	++	++	+/-	+/-	+/-	-
**33A-3F3**	++	+++	+	+++	++	+/-	-
**33A-3H1**	+	+	+/-	+/-	+/-	+	+/-
**36S-C11**	+	++	+	+	+/-	+/-	-
**1D12**	++++	+++	+++	+/-	+/-	++	+
**1F11**	++++	++++	+++	+/-	+/-	++	+/-
**2H6**	++++	++++	+++	+/-	+/-	+++	+
**4B10**	++	++	++	+	-	+++	+
**4C5**	++++	++++	+++	+	+	+	+
**9C10**	++++	++++	++++	+/-	+/-	+++	+

Semi-qualitative grading of the reactivity for each antibody for αS inclusion pathology in formalin-fixed substantia nigra (SN) of patients with Parkinson’s disease (PD) or dementia with Lewy Bodies (DLB), the cingulate cortex (Cing) of patients with DLB, or the pons and cerebellum (CB) of patients with multiple system atrophy (MSA). Similar grading of staining of αS pathological inclusions in formalin- or ethanol fixed M83 mouse tissues. The findings are summarized as (-) negative, (+/-) very weak, (+) weak, (++) modest, (+++) strong and (++++) very strong.

**Fig 9:**
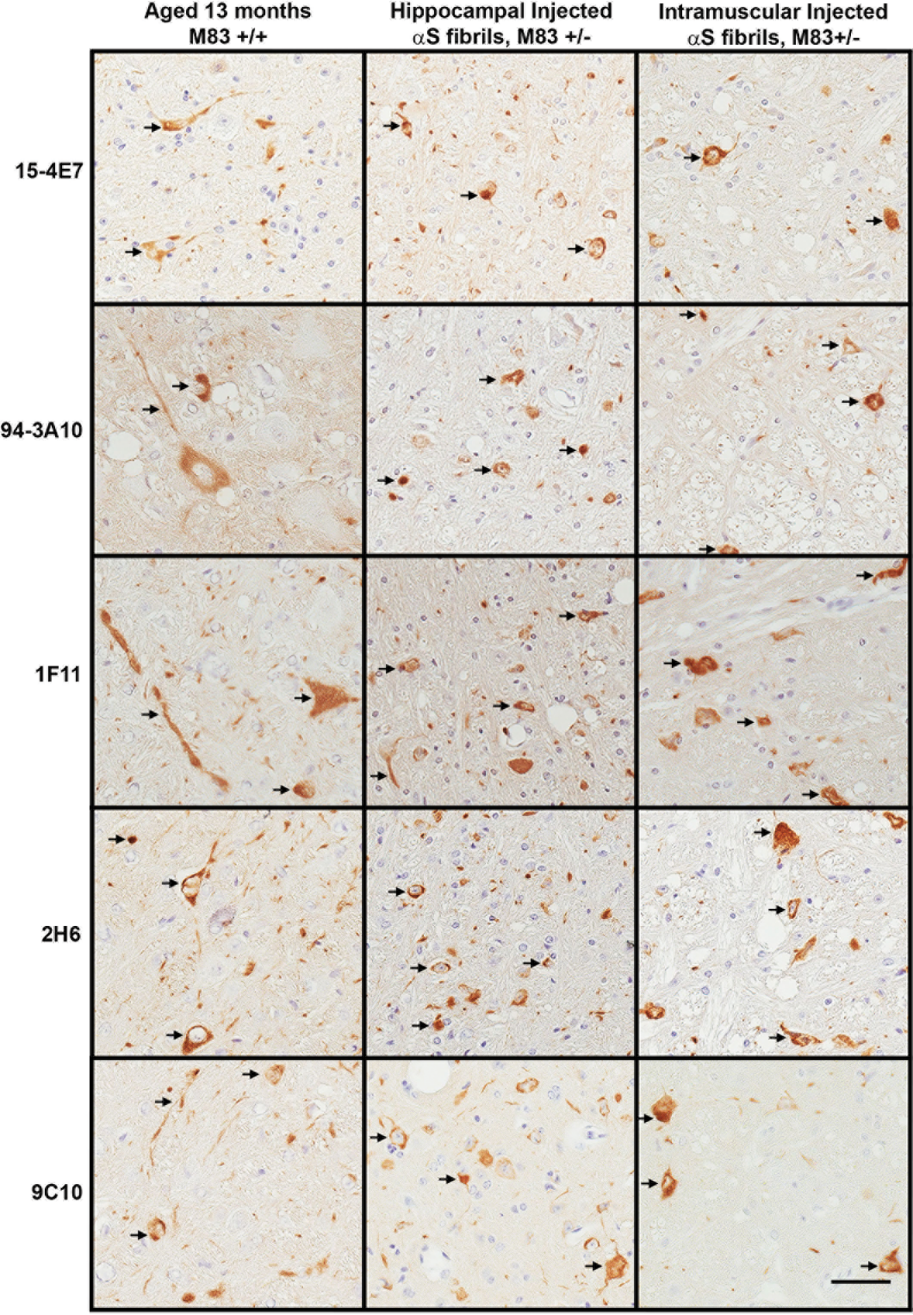
Immunohistochemical analysis of αS inclusion pathology using various M83 models of synucleinopathies and novel αS antibodies. Representative images of αS pathology in the midbrain of a 13 month old motor impaired M83^+/+^ mouse, the midbrain of a M83^+/-^ mouse hippocampally injected with recombinant human αS fibrils to induce αS inclusion pathology, and the brainstem of a M83^+/-^ mouse intramuscularly injected with mouse αS fibrils resulting in a prion-like peripheral to CNS transmission and induction of αS pathology. Tissue sections were stained with each indicated antibody and developed with DAB as described in “Material and Methods” and counterstained with hematoxylin. Arrows indicate αS inclusion pathology. Bar = 50 μm.

Next, we assessed the ability our antibodies to detect αS inclusion pathology in various models (age-dependent or seeding-induced) of synucleinopathy. These included aged M83^+/+^ transgenic mice, M83^+/-^ transgenic mice injected with preformed αS fibrils in the hippocampus, and M83^+/-^ transgenic mice injected with preformed αS fibrils in hind limb muscle resulting in prion-like transmission and induction of CNS αS inclusion pathology (Table 1). These mouse models develop wide spread midbrain, brainstem and spinal cord αS pathology, with increased burden in the cortex and hippocampus of hippocampally injected mice. Since fixation can also affect the ability to retrieve antigen staining [31], we also compared staining patterns in mouse nervous tissue fixed with ethanol or formalin (Table 3). Generally, ethanol fixation does not result in direct covalent modification and cross-linking of the antigen as occurs in formalin fixation that can abrogate the epitopes, but it is less efficient at maintaining unique protein conformations. For most antibodies ethanol fixation produced better staining (Table 3). Some antibodies, such as 2H6 and 4B10, showed differential immunohistochemical staining patterns in mouse nervous tissue depending on the method of fixation, while others, such as 15-4E7 and 94-3A10, recognized αS inclusions irrespective of fixation method. Syn 6, 15-4E7, 94-3A10, and the N-terminal antibodies, produced the best staining of αS inclusion pathology in all 3 models of synucleinopathy assessed (Table 3 and Fig 9).

## Discussion

Herein, we developed and characterized a panel of αS antibodies for the purpose of expanding the available toolkit to assess unique αS associated with specific pathologies. These antibodies were first characterized for possible cross-reactivity with other proteins including other synuclein proteins. Immunization with recombinant proteins demonstrated that the amino acid region 89–140 in αS is more immunogenic than the rest of the protein since all monoclonal antibodies generated have epitopes against this region. While the immunogen used for most of the antibodies generated was an αS construct lacking the first 20 N-terminal residues, no antibodies were found to target residues 21 to 89, a region of αS containing a portion of the N-terminal amphipathic domain and a portion of the hydrophobic core [1,32], indicating a region of reduced immunogenicity. However, using a synthetic peptide approach we were able to generate monoclonal antibodies to amino-terminal residues 2–21. Of note, antibodies 1F11 and 4C5 were determined to be of the IgA isotype, a rare isotype when not specifically isolated that could prove useful in future bioassays.

None of the antibodies with epitopes within amino acid region 89–140 in αS cross-reacted with γS due to the low homology between these 2 proteins in this region. Some of these antibodies could cross-react with βS due to the greater homology. Given the greater homology of synuclein proteins at the N-terminal, all the amino-terminal antibodies could react with βS, and some with γS. Furthermore, due to amino acid differences between mouse and human αS, several of the monoclonal antibodies that recognize epitopes in the C-terminal of αS are specific for human αS. For example, all of the antibodies with epitopes within residues 120–125 such as 15-2C8, 15-4D5, and 33A-2A1 are human αS specific due to ^121^DN^122^ to ^121^GS^122^ amino acid difference in human versus mouse αS. Furthermore, using total mouse brain homogenates, including αS null mice, we showed that most of the new αS antibodies are specific for αS or the other synuclein proteins that some antibodies react with.

The immunocytochemical studies revealed significant difference in the overall staining profiles of different types of αS pathological inclusions when compared across the series of αS antibodies. Although the amino-terminal antibodies could strongly reveal both classic nigral LBs and cortical LBs, they are not as efficient at detecting GCIs. These findings are consistent with previously reported unique immunoreactive difference between LBs and GCIs [8]. The molecular changes responsible to these distinctions are not fully understood, but besides both types of inclusions being present in different cells types, the ultrastructure of αS fibrils in GCIs is distinct that those in LBs [2,6,10]. For example, the fibrils that comprise in GCIs can have wider and a more tubular structure compared to the filamentous αS that comprise in LBs [2,6,10]. This ultrastructural variances in addition to more subtle molecular conformational differences would be consistent with divergent immune-labeling profiles.

Generally, antibody staining of nigral LBs was more robust than the labeling for cortical LBs. Classic nigral LBs have a well-defined morphological profile with a core and halo versus the more irregular shape of cortical LBs. The reason for these different types of αS protein aggregates are still hypothetical, but the differences in staining profile across our large array of antibodies further suggest that the αS in both types of inclusions is molecularly distinct. This could be due to differences in pathologically relevant post-translation modifications and/or conformational differences, that can potentially contribute to protein strain properties of αS inclusions present in different synucleinopathies. However, antibody 94-3A10 and the amino-terminal antibodies demonstrated the most robust labelling for both classical and cortical LBs. Furthermore, when staining GCIs antibody 94-3A10 produced robust staining indicating that this antibody is less influenced by αS inclusion-specific alterations.

Interestingly, antibodies 15-4E7, 94-3A10, and some of the N-terminal antibodies were the most efficient at targeting αS pathology in the mouse models observed. 15-4E7 and 94-3A10 target an epitope in residues 130–140, and while 15-4E7 weakly stained αS pathology in human cases of synucleinopathy, it excelled in targeting αS deposits in each investigated mouse model. 15-4E7 could only weakly react with βS by ELISA, in comparison to 94-3A10 which could bind it more efficiently. Residues 130 and 132 differ between αS and βS, possibly indicating this local region is involved in 15-4E7 binding while 94-3A10 more likely targets the extreme C-terminus. 94-3A10 therefore represents our most efficient αS pathology specific antibody and thus might be the most useful for general immunotherapy.

Immunotherapy has recently been established as a viable method for both reducing protein aggregate burden and associated behavioral deficits in several mouse models of α-synucleinopathy [33–47]. These reports have led to both active and passive immunotherapy clinical trials. For example, the biotechnology company AFFIRiS AG reported using an active immunization protocol with small peptide fragments of the C-terminus of αS, entitled AFFITOPE®, and has completed Phase 1 trials in addition to a follow up “boost” study with the results of the long-term safety and clinical effects to be released in 2017 (clinicaltrials.gov identifier: NCT02216188) [48]. While active immunization, or the elicitation of an adaptive antibody immune response to an administered immunogen, has the advantages of costing considerably less and producing long lasting antibody titers, it unfortunately has several disadvantages. The wide variability in potential targetable epitopes is reduced by the approach AFFIRiS AG utilizes but on the other hand the potential off target effects of a polyclonal active immunization approach cannot be fully accounted for. There are significant uncertainties and risks associated with active immunization as both positive and negative outcomes, such as T-cell activation, have been suggested [43,49,50]. In addition, patient immune system variability has been shown to produce differential immune responses, possibly as a result of age-induced immune deficits [51–53], which has significant implications since neurodegenerative patients tend to be elderly.

Passive immunotherapy, or the administration of previously generated antibodies, has the advantage of producing an immediate immune response, with immense target specificity since antibodies administered are monoclonal, but its viability as a potential therapy is diminished by the high cost of antibody production and the temporary immunity it produces. Nevertheless, Prothena Biosciences Inc. in collaboration with the Swiss healthcare company Roche, developed αS antibodies and utilizing a passive immunotherapy protocol has also completed Phase 1 and 1b trials with Phase 2 expected to begin in 2017 (clinicaltrials.gov identifier: NCT02157714) [54]. Additionally, BioArctic AB has generated antibodies selective for oligomeric and protofibril forms of αS and begun preliminary in-man clinical trials (http://www.bioarctic.se/parkinson)[34,47].

Since antibody 94-3A10 is capable of selectively targeting αS inclusion pathology across the investigated disease states it could be considered the model for a global approach regarding passive immunotherapy. αS residues 130–140 might be present in a common pathological state such that differences that lead to the differential deposition of αS in synucleinopathies are not present in this region. However, given that the αS C-terminus can be cleaved during αS pathology progression [17,18,55–57], and that C-terminal cleavage products preferentially promote αS aggregation likely promoting intracellular and intercellular prion-like transmission of αS [20,57,58], an antibody directed to an epitope located further towards the middle of the molecule and present even after C-terminal truncation may be more advantageous. With this in mind, while 94-3A10 may have proven to be the most robust antibody for identifying pathology through bioassays, it may target a region that does not play a key role in prion-like transmission, and instead this region, through protective cellular stress mechanisms or other aggregative processes, is sequestered into the noticeable deposits seen through immunohistochemistry.

We have generated and demonstrated the specificity of a series of new αS monoclonal antibodies recognizing distinct epitopes. Using these reagents we demonstrate that αS inclusion pathology in different types of synucleinopathies and even with different brain regions display distinct immuno-reactive properties. These antibodies will be useful in future studies of αS pathology progression involving specific biochemically altered and cleaved αS species. These antibodies also provides important tools to assess which region of αS may be the most effective target for passive immunization in synucleinopathy immunotherapy. These issues will be investigated in future studies utilizing preclinical models.
